# Being Friends with or Rejected by Classmates: Aggression Toward Same- and Cross-Ethnic Peers

**DOI:** 10.1007/s10964-019-01173-1

**Published:** 2019-12-06

**Authors:** Marianne Hooijsma, Gijs Huitsing, Jan Kornelis Dijkstra, Andreas Flache, René Veenstra

**Affiliations:** Department of Sociology and Interuniversity Center for Social Science Theory and Methodology (ICS), University of Groningen, Groningen, the Netherlands

**Keywords:** Aggression, Ethnicity, Friendship, Rejection, Adolescence

## Abstract

Whereas previous research suggests that adolescents’ aggressive behavior in itself does not highlight ethnic boundaries, it remains unclear whether classmates’ responses to same- and cross-ethnic aggression strengthen ethnic boundaries. This study examined how adolescents’ aggression toward same- and cross-ethnic peers relates to the positive (friendship) and negative (rejection) relationship nominations they receive from same- and cross-ethnic classmates. Cross-sectional peer nomination data on 917 Dutch and 125 Turkish adolescents in 56 secondary schools were analyzed (mean age = 14.9 year; 51.4% boys). Adolescents received more friendship nominations from same-ethnic than from cross-ethnic classmates, but were not more rejected by cross-ethnic than same-ethnic classmates. Multilevel Poisson and negative binomial regression models showed that, irrespective of aggressor’s ethnic background, adolescents’ aggressive behavior was related to rejection by classmates from the ethnic group that was the target of aggression and to being befriended by classmates from the ethnic group that was not the target of aggression. Specifically, both Dutch and Turkish adolescents who were aggressive toward Dutch peers were rejected by Dutch classmates and befriended by Turkish classmates and vice versa. These findings suggest that classmates’ positive and negative responses to adolescents are related to adolescents’ aggressive behavior based on the ethnic background of the victim, not on the ethnic background of the aggressor. This suggests that integration between ethnic groups in schools relates to aggression in general, not only cross-ethnic aggression.

## Introduction

In the context of ethnically diverse classrooms, interethnic aggression may be perceived as behavior that reinforces ethnic boundaries. Previous research has shown that although ethnic groups might slightly differ in the extent to which they behave aggressively, same- and cross-ethnic aggression was equally common (Tolsma et al. 2013; Vitoroulis and Vaillancourt 2018). Whereas this suggests that adolescents’ aggressive behavior in itself may not highlight ethnic boundaries, it remains unclear how classmates respond to adolescents’ same- and cross-ethnic aggression and, consequently, whether aggressive behavior might indirectly affect ethnic boundaries in classrooms. In this study, it is questioned how adolescents’ aggressive behavior toward same- and cross-ethnic peers relates to the positive (friendship) and negative (rejection) relationship nominations they receive from their classmates. Do these responses to adolescents relate to their aggressive behavior in ways that reflect or emphasize ethnic boundaries in the classroom?

### The Role of Ethnicity in Friendship and Rejection

In the context of ethnic heterogeneity, social identity theory proposes that through identification with the in-group (i.e., same-ethnic peers), individuals have the tendency to evaluate the in-group positively (Tajfel and Turner 1979). Aiming to achieve a positive social identity, individuals categorize their environment into groups and, specifically, compare their own group to other groups (i.e., cross-ethnic peers). In this process of differentiating the in-group from out-groups, individuals develop in-group favoritism, referring to individuals’ preference to affiliate with others whom they perceive to belong to their in-group (Tajfel and Turner 1979). Consequently, individuals are more likely to feel connected to in-group members than to out-group members. Positive peer relationships, such as friendships, are more likely between same-ethnic peers (Rivera et al. 2010). Similarity in, for example, ethnicity, enhances both agreement and understanding and makes the other’s behavior predictable (Hamm 2000; Ibarra 1992). Same-ethnic peers have a similar cultural background which relates to more similar norms and values compared to cross-ethnic peers. This similarity facilitates the initiation and maintenance of positive peer relationships. As ethnicity is an important characteristic in categorizing groups in adolescence (Boda and Néray 2015; Leszczensky and Pink 2015), adolescents are expected to favor their same-ethnic peers over their cross-ethnic peers.

Whereas several studies have found evidence for ethnic boundaries and segregation in adolescents’ positive peer relationships (Boda and Néray 2015; Leszczensky and Pink 2015; Stark and Flache 2012), less is known about the role of ethnicity in adolescents’ negative peer relationships. In line with social identity theory (Tajfel and Turner 1979), group categorization and comparison of the in-group to out-groups may lead to more negative evaluations of out-group members. Categorizing groups along ethnic boundaries signals differences between groups, for example in terms of cultural norms and practices (Strohmeier et al. 2008). Consequently, negative peer relationships can be expected to be more likely between adolescents of different ethnic groups.

### Aggression Toward Peers of Different Ethnic Groups

Aggressive behavior toward peers is a powerful means to gain status in the classroom (Sijtsema et al. 2009). Moreover, when used strategically, aggressive behavior poses a minimum risk for loss of connectedness by significant peers, for instance by selecting victims, both same- and cross-ethnic, who are rejected by the aggressor’s significant others (Veenstra et al. 2010). Victims of aggression are, however, at risk of losing both status and connectedness as they are likely to be avoided and less accepted by classmates (Van der Ploeg et al. 2015). Classmates may not want to affiliate with victims as this puts them at risk of being victimized as well (Sentse et al. 2013). Given the assumption that individuals act as a response to the awareness of factors that foster or threaten their goal pursuit by liking the former and disliking the latter (Lindenberg 2001), aggressors are likely to be rejected by their victims because they threaten their victim’s goal pursuit.

Moreover, as victims’ in-group members may feel threatened by the aggressor as well, aggressive behavior is also likely to relate to rejection by victims’ in-group peers. Among early adolescents it has been found that victims’ same-sex peers rejected the victims’ bullies (Veenstra et al. 2010). As peers who do not belong to the victim’s in-group are less likely to be threatened by the behavior of the aggressor, aggressors were not rejected by their victims’ cross-sex peers (Veenstra et al. 2010). Similarly, it may be expected that aggression toward members of a specific ethnic group in the classroom is only related to rejection by classmates from that specific ethnic group, and not to rejection by classmates from other groups.

Although it can be expected that adolescents would generally tend to evaluate aggression toward same-ethnic peers negatively, it can also be argued that this relation is stronger if the aggressor is same-ethnic than if the aggressor is cross-ethnic. In the context of differentiating and comparing of the in-group to out-groups, the most common pattern would be that aggression by cross-ethnic peers may be perceived as norm-conforming behavior. Aggression to same-ethnic peers, however, is likely to be perceived as norm-deviating behavior. Specifically, aggression by an in-group member toward one’s in-group can be seen as a form of betrayal of the in-group. Adolescents who feel betrayed by their significant peers distrust and avoid interactions with these peers. In an experimental setting, it was indeed found that individuals negatively evaluate in-group members who are disloyal to their group (Travaglino et al. 2014).

Whereas cross-ethnic aggression is expected to be related to rejection by the cross-ethnic group as the target of aggression, it can be argued that cross-ethnic aggression is also related to positive evaluations by same-ethnic peers. Next to social needs, such as achieving status and being connected, adolescents have individual psychological needs, such as developing a positive social identity. In order to achieve a positive social identity, individuals tend to differentiate the in-group from and compare the in-group to out-groups in ways to devaluate out-groups (Tajfel and Turner 1979). Aggressive behavior toward out-group peers is an example of a way to devaluate the out-group. Aggressive behavior toward out-groups may highlight differences between groups and consequently benefit in-group members’ development of a positive social identity. Given that individuals tend to like characteristics that foster their goal pursuit (Lindenberg 2001), such as achieving a positive social identity, it can be expected that cross-ethnic aggression would be related to positive evaluations of same-ethnic classmates of the aggressor.

## Current Study

As previous research has shown that cross-ethnic aggression was not more likely than same-ethnic aggression (Tolsma et al. 2013), aggressive behavior in itself may not highlight ethnic boundaries. It is unclear, however, whether classmates’ responses to adolescents’ same- and cross-ethnic aggression emphasize ethnic boundaries in the classroom. In the current study, it is first examined how the positive and negative relationship nominations adolescents receive from same- and cross-ethnic classmates differ by their ethnic background. In the process of differentiating the in-group from out-groups to achieve a positive social identity, individuals tend to develop a preference for affiliating with in-group members over out-group members. Moreover, similarity between peers enhances agreement, understanding, and predictability in relationships. Consequently, it is hypothesized (*H1*) that adolescents would receive relatively more friendship nominations from same-ethnic classmates than cross-ethnic classmates. Furthermore, comparing the in-group to out-groups may lead to more negative evaluations of out-group members. Therefore, it is hypothesized (*H2*) that adolescents would be rejected by relatively more cross-ethnic classmates than same-ethnic classmates.

Second, the current study examined how adolescents’ same- and cross-ethnic aggressive behavior relates to the positive and negative relationship nominations they receive from their same- and cross-ethnic classmates. As adolescents’ aggressive behavior threatens the goal pursuit of their victims and their victims’ in-group classmates, but not that of the victims’ out-group classmates, it is hypothesized (*H3*) that aggressive adolescents would be rejected by classmates from the ethnic group to whom the victim belongs, but not by classmates from other ethnic groups in the classroom. Aggression toward same-ethnic classmates may be perceived as norm-deviating behavior and betrayal. Consequently, it is hypothesized (*H4*) that adolescents’ aggression toward same-ethnic peers would relate more to rejection by same-ethnic classmates than aggression toward cross-ethnic peers. Finally, aggression toward cross-ethnic peers may be a way to devaluate the out-group, benefiting the development of same-ethnic peers’ positive social identity. Therefore, it is hypothesized (*H5*) that adolescents who are aggressive toward cross-ethnic peers would receive more friendship nominations from same-ethnic classmates than cross-ethnic adolescents who are aggressive toward this cross-ethnic group.

This study examined classmates’ response to adolescents’ aggressive behavior toward same- and cross-ethnic peers in secondary school classrooms in the Netherlands. Specifically, this study examined these relationships among Dutch and Turkish adolescents. As part of labor migration, the first Turkish immigrants came to the Netherlands in the 1960s. Although the Dutch society’s ethnic composition is changing, with immigrants from more diverse ethnic backgrounds coming to the Netherlands, immigrants with a Turkish background remain to form the largest ethnic minority group in the Netherlands and in Dutch secondary schools (Statistics Netherlands 2018).

The extent to which adolescents are aggressive toward, as well as befriended or rejected by, Dutch or Turkish classmates depends partially on the opportunity structure, i.e., whether adolescents have Dutch or Turkish classmates. Similarly, the extent to which adolescents are befriended or rejected by classmates may differ between boys and girls. Previous research has, for example, shown that boys are more aggressive toward and more rejected by classmates (Veenstra et al. 2010). In examining adolescents’ aggressive behavior and the relationship nominations they receive from classmates, this study therefore controls for the number of Dutch and Turkish classmates as well as adolescents’ sex.

## Methods

### Procedure

Data was collected in the Netherlands for the first wave (2010/2011) of the Children of Immigrants Longitudinal Survey in Four European Countries (CILS4EU; Kalter et al. 2016). The CILS4EU project focuses on the intergenerational integration of children of immigrants. Early adolescent immigrants and their majority peers around the age of 14 to 15 (3rd grade of secondary school) were the target population. A three-stage sampling method was used.

First, secondary schools were selected based on a probability proportional to size as well as the proportion of students with an immigrant background (referring to students who were themselves, or had at least one parent who was, born in a non-Western country). Schools were excluded from the school sample when the total number of students was less than 2% of the total target population and the target classroom size was smaller than one quarter of the average classroom size, or when it were special schools for students with cognitive, emotional, or physical disabilities. 34.9% of the 109 originally targeted schools participated. To increase the number of participating schools, a replacement strategy was implemented in which replacement schools were selected which matched the non-participating schools based on proportion of students with an immigrant background and school type. After this replacement strategy was implemented, 100 schools participated. Second, in most schools, two classrooms were selected. In schools with more than 60% immigrant students, as many classrooms as possible were selected to increase the number of participating immigrants. Of the selected classrooms, 94.5% (222 classrooms) participated in the study. Third, only students not being able to respond to the questionnaire in the language of the host country were excluded from the sample. Exclusions within schools were negligible. In total, 91.1% (4363 students) of the selected students participated in the study.

The CILS4EU survey was administered using paper-and-pencil questionnaires. Extensively trained students assistants were selected to administer the survey. In order to ensure privacy, questionnaires were identified with a unique ID number, which was linked to the specific student on a separate class list.

### Sample

This study investigated the relation between aggression toward and being befriended and rejected by classmates specifically for the two largest ethnic groups in the Dutch CILS4EU data, namely non-immigrant Dutch adolescents and adolescents with an immigrant background from Turkish origin. Of these adolescents, those who had at least one Dutch and one Turkish classmate were selected. This resulted in a sample of 1042 adolescents (917 Dutch and 125 Turkish adolescents) in 85 classes in 56 schools. Adolescents in the sample were on average 14.9 years old (*SD* = 6.8 months) and there were about as many boys (51.4%) as girls. On average, Dutch adolescents’ socio-economic background was higher than that of Turkish adolescents (based on the international socio-economic index of occupational status, ISEI, ranging from 0 to 100; Ganzeboom et al. 1992; average ISEI for Dutch adolescents = 51.8, average ISEI for Turkish adolescents = 35.6). Only one participant in the sample had a missing value on one of the study variables. For this Turkish adolescent, information on sex was missing. As the exclusion of this participant will not likely influence the results, it was decided to exclude the participant from the analyses.

### Measures

#### Immigrant background

Immigrant background was assessed using information on students’, their parents’, and their grandparents’ country of birth and country of origin as provided by the student as well as their parents. Students who themselves, their parents, and grandparents were born in the Netherlands, were classified as ‘Dutch’. Students who themselves, their parents or grandparents were born outside of the Netherlands were classified as having an immigrant background. A bottom-up approach was used to define immigrant adolescents’ ethnic origin in which information at the grandparent level was used first, followed by information about the parents and the adolescent. For the grandparent and parent levels, two decision rules were applied to define adolescents’ ethnic origin. First, the majority rule indicated that if the majority of (grand)parents was born in a certain country, this information was used to define adolescent’s ethnic origin. Second, the priority rule indicated that if there was no majority, priority was given to the country of birth of the (maternal grand)mother (for details on the definition of adolescents’ ethnic origin: Dollmann et al. 2014).

#### Friendship

Participants responded to the question: “Who are your best friends?”. They received a list of names and random identification numbers of all of their classmates (also non-participating classmates) and were asked to nominate up to five classmates as their best friends. To measure the extent to which students were befriended by their Dutch and Turkish classmates, the number of nominations for best friends they received from each of the groups was calculated. Furthermore, the proportion of nominations for best friends students received from each of the groups compared to the total number of nominations they could have received from these groups was calculated.

#### Rejection

Participants were also asked to nominate classmates they did not want to sit close to (“Who would you not want to sit by?”), which is taken as a proxy for rejection. Participants could nominate up to five classmates. To measure how often students were rejected by their Dutch and Turkish classmates, the number and proportion of rejection nominations students received from each of these groups were calculated.

#### Aggression

Participants were asked to nominate an unlimited number of classmates they perceived as being mean to them (“Who is sometimes mean to you?”). To measure students’ aggression toward their Dutch and Turkish classmates, the number and proportion of nominations students received from each of these groups were calculated.

#### Control variables

Self-reported *sex* was included to control for possible sex differences in being befriended or rejected by classmates (girls were coded as 0 and boys were coded as 1). In addition, to control for the *availability of same- and cross-ethnic classmates*, the number of Dutch and Turkish classmates adolescents have were included in the analyses.

### Analytical Strategy

To account for the availability of Dutch and Turkish classmates, proportions of nominations are discussed in the descriptive statistics. In the confirmatory analyses, the absolute number of nominations is used while accounting for the number of Dutch and Turkish classmates.

#### Main analyses

Poisson regression models in Stata version 15.1 (StataCorp 2017) were used to examine the relation between aggression, friendship, and rejection because of the non-negative count characteristic of the dependent variables (Cameron and Trivedi 2013). Given that adolescents are more likely to be befriended or rejected by Dutch or Turkish classmates in classrooms with, respectively, more Dutch or Turkish students, the number of Dutch or Turkish classmates was used as an exposure variable, indicating the number of nominations an adolescent could have received. That is, the number of Dutch or Turkish classmates was used as on offset to account for opportunity differences (see Long and Freese 2006). Furthermore, robust standard errors for the parameter estimates were examined to control for mild violation of underlying assumptions (Cameron and Trivedi 2009).

Before conducting the regression models, it was checked whether the dependent variables’ variance exceeded the mean, i.e., whether there was an issue of overdispersion. This was checked by comparing the full regression model using a regular Poisson distribution to a model using a negative binomial distribution, which accounts for overdispersion. The difference between the model was tested using a likelihood ratio test of the overdispersion parameter alpha. For being befriended by Dutch or Turkish classmates, overdispersion was not found to be an issue (*Χ*^*2*^*=* 0.94, *p* = 0.17; negative binomial models could not be estimated for being befriended by Turkish classmates due to convergence problems). For being rejected by Dutch or Turkish classmates, however, overdispersion was found (*Χ*^*2*^ = 363.37, *p* < 0.001; *Χ*^*2*^ = 8.75, *p* = 0.002). Therefore, Poisson regression models were used to examine the extent to which adolescents were befriended by Dutch and Turkish classmates, whereas for their rejection by Dutch and Turkish classmates negative binomial regression models were used.

Students were nested in classrooms which were nested in schools. Given the low number of selected classrooms in each school, school-level variation was comparable to classroom-level variation. The intraclass correlation at the classroom-level was high for being befriended (0.34 and 0.27) and rejected by classmates (0.13 and 0.27) and the design effect was larger than 2 indicating the importance of accounting for the two-level structure in our data (Muthen and Satorra 1995). Moreover, likelihood ratio tests comparing the full regression model using a single-level model to a two-level model showed that the two-level Poisson regression model was more appropriate to examine the extent to which adolescents received friendship nominations from Dutch and Turkish classmates (*Χ*^2^*=* 28.66, *p* < 0.001; *Χ*^2^*=* 4.67, *p* = 0.02) and the two-level negative binomial models were more appropriate to examine adolescents’ rejection by Dutch and Turkish classmates (*Χ*^2^*=* 15.15, *p* < 0.001; *Χ*^2^*=* 11.58, *p* < 0.001). To account for the two-level structure, multilevel Poisson and negative binomial regression models were used.

The regression analyses were conducted separately for being befriended by and rejected by either Dutch or Turkish classmates as the dependent variables. Each regression analysis consisted of three models. In Model 1, we added a dummy indicating adolescents’ ethnic background to examine whether being befriended by (*hypothesis 1*) or rejected by (*hypothesis 2*) classmates differed between cross- and same-ethnic peers. In Model 2, we added aggression toward Dutch and Turkish peers as independent variables to examine the association between aggression toward each of these groups and being befriended or rejected by (*hypothesis 3*) classmates from each of these groups. In Model 3, we added interactions between adolescents’ ethnic background and their aggression toward Dutch and Turkish peers to examine whether the relation between aggression toward each ethnic group and being rejected (*hypothesis 4*) or befriended by (*hypothesis 5*) classmates differed between cross- and same-ethnic peers. All models controlled for adolescents’ sex and availability of Dutch or Turkish classmates. All continuous independent variables were grand-mean centered.

#### Additional analyses

In addition to the analyses among Dutch and Turkish adolescents, analyses were conducted for two other immigrant groups: Dutch and Moroccan (810 Dutch and 117 Moroccan adolescents in 75 classes), and Dutch and Surinamese (1099 Dutch and 121 Surinamese adolescents in 91 classes) adolescents. The measures and analytical strategy for these additional analyses are similar to the analyses for Dutch and Turkish adolescents. Results of the additional analyses can be found in Appendix 1.

#### Disclosure statement

All information regarding the selection of the sample, data exclusions, manipulations, and measures in the study are reported.

## Results

### Descriptive Results

Table 1 shows that overall, Dutch and Turkish adolescents did not differ much in their general aggression toward (*M*_Dutch_ = 0.02, *M*_Turkish_ = 0.04), being befriended (*M*_Dutch_ = 0.16, *M*_Turkish_ = 0.15), and rejected (*M*_Dutch_ = 0.10, *M*_Turkish_ = 0.12) by Dutch and Turkish classmates. Moreover, adolescents were not more aggressive toward same- or cross-ethnic peers (Dutch toward Dutch: *M=* 0.03; Dutch toward Turkish: *M* = 0.02; Turkish toward Dutch: *M* = 0.05; Turkish toward Turkish: *M* = 0.04), and did not receive more proportions of nominations for rejection by cross-ethnic than same-ethnic classmates (Dutch by Dutch: *M=* 0.11; Dutch by Turkish: *M* = 0.10; Turkish by Dutch: *M* = 0.13; Turkish by Turkish: *M* = 0.08). For friendships, adolescents received higher proportions of nominations from same-ethnic classmates than from cross-ethnic classmates (Dutch to Dutch: *M* = 0.17; Dutch to Turkish: *M* = 0.12; Turkish to Dutch: *M* = 0.09; Turkish to Turkish: *M* = 0.33).

**Table 1:** Descriptive statistics on aggression, friendships, and rejection for Dutch and Turkish adolescents

	Aggression toward		Befriended by		Rejected by	
	*M* (SD)	Max. number of nominations	*M* (SD)	Max. number of nominations	*M* (SD)	Max. number of nominations
Dutch adolescent	0.02 (0.05)	10	0.16 (0.10)	11	0.10 (0.14)	18
Dutch classmates	0.03 (0.07)	5	0.17 (0.13)	10	0.11 (0.15)	16
Turkish classmates	0.02 (0.13)	2	0.12 (0.31)	3	0.10 (0.29)	5
Turkish adolescent	0.04 (0.06)	6	0.15 (0.12)	7	0.12 (0.13)	17
Dutch classmates	0.05 (0.13)	5	0.09 (0.19)	4	0.13 (0.24)	9
Turkish classmates	0.04 (0.16)	2	0.33 (0.37)	4	0.08 (0.20)	2

Means and standard deviations are based on proportion of nominations. Minimum number of nominations is 0 in all cases

Table 2 presents correlations between aggression toward and being befriended and rejected by the two ethnic groups. The table shows that adolescents who were aggressive toward one of the groups were also more likely to be aggressive toward peers from the other ethnic group (*r* = 0.16, *p* < 0.001). Similarly, adolescents who were rejected by classmates from one group were also likely to be rejected by classmates from the other group (*r* = 0.25, *p* < 0.001). No association was found between being befriended by the two groups (*r* = −0.03, *p* = 0.28).

**Table 2:** Correlations

	1.	2.	3.	4.	5.	6.
Aggression toward						
1. Dutch						
2. Turkish	0.16**					
Befriended by						
3. Dutch	−0.04	0.07*				
4. Turkish	0.15**	0.05	−0.03			
Rejected by						
5. Dutch	0.40**	0.06	−0.16**	0.03		
6. Turkish	0.02	0.20**	−0.04	−0.10*	0.25**	

Correlations are calculated using proportion of nominations

*p < 0.05; **p < 0.001

Descriptively it was found that aggression toward one of the groups was positively related to rejection by classmates from this specific group (*r*_Dutch_ = 0.40, *p* < 0.001; *r*_Turkish_ = 0.20, *p* < 0.001) but not to rejection by classmates from the other group. Also, although with smaller correlations, aggression toward one group related positively to being befriended by classmates from the other group: aggression toward Dutch related positively to being befriended by Turkish classmates (*r* = 0.15, *p* < 0.001) and aggression toward Turkish related positively to being befriended by Dutch classmates (*r* = 0.07, *p* = 0.04).

### The Role of Ethnicity in Friendship and Rejection

In line with hypothesis 1 (adolescents would receive relatively more friendship nominations from same-ethnic classmates than cross-ethnic classmates), it was found in the multilevel Poisson regression models that Dutch adolescents were befriended by more Dutch classmates (*PE* = 0.77, *p* < 0.001) and befriended by fewer Turkish classmates (*PE* = −1.02, *p* < 0.001) than Turkish adolescents, see Model 1 in Table 3 (left panel for being befriended by Dutch classmates, right panel for being befriended by Turkish classmates).

**Table 3:** Results of multiple multilevel Poisson regression models for friendship

	Befriended by Dutch									Befriended by Turkish								
	Model 1			Model 2			Model 3			Model 1			Model 2			Model 3		
Parameter	*PE*	*SE*	*p*	*PE*	*SE*	*p*	*PE*	*SE*	*p*	*PE*	*SE*	*p*	*PE*	*SE*	*p*	*PE*	*SE*	*p*
Constant	−2.58	0.13		−2.59	0.13		−2.59	0.14		−1.17	0.18		−1.18	0.17		−1.20	0.18	
Ethnic background (Dutch = 1)	0.77	0.13	<0.001	0.78	0.14	<0.001	0.78	0.14	<0.001	−1.02	0.19	<0.001	−1.01	0.19	<0.001	−0.98	0.20	<0.001
Aggression toward																		
Dutch				−0.04	0.03	0.18	−0.10	0.08	0.19				0.24	0.07	0.001	0.30	0.09	0.001
*Ethnic background (Dutch = 1)							0.06	0.08	0.42							−0.07	0.14	0.59
Turkish				0.25	0.11	0.02	0.26	0.26	0.32				0.04	0.20	0.84	0.20	0.21	0.34
*Ethnic background (Dutch = 1)							−0.02	0.28	0.95							−0.63	0.57	0.28
Sex (boy = 1)	0.05	0.06	0.39	0.05	0.06	0.39	0.05	0.06	0.39	−0.09	0.16	0.59	−0.12	0.16	0.46	−0.12	0.16	0.46
Number of Dutch classmates	Exp.			Exp.			Exp.			−0.01	0.02	0.65	−0.01	0.02	0.46	−0.01	0.02	0.43
Number of Turkish classmates	−0.02	0.03	0.41	−0.03	0.03	0.30	−0.03	0.03	0.30	Exp.			Exp.			Exp.		
Variance classroom	0.05	0.03		0.05	0.03		0.05	0.03		0.11	0.06		0.10	0.06		0.09	0.05	
Log likelihood	−1698.40			−1696.21			−1696.13			−586.70			−582.80			−581.91		

The number of Dutch or Turkish classmates was used as an exposure variable to account for opportunity differences

*It indicates an interaction effect

Similarly, Models 1 in Table 4 were used to test hypothesis 2 that adolescents would be rejected by relatively more cross-ethnic classmates than same-ethnic classmates. In contrast to the hypothesis, no evidence was found that Dutch adolescents were rejected by fewer Dutch classmates than Turkish adolescents (*PE* = −0.24, *p* = 0.20). In line with the hypothesis, however, Dutch adolescents were rejected by slightly more Turkish classmates than Turkish adolescents (*PE* = 0.67, *p* = 0.05). Thus, partial support was found for hypothesis 2.

**Table 4:** Results of multiple multilevel negative binomial regression models for rejection

	Rejected by Dutch									Rejected by Turkish								
	Model 1			Model 2			Model 3			Model 1			Model 2			Model 3		
Parameter	*PE*	*SE*	*p*	*PE*	*SE*	*p*	*PE*	*SE*	*p*	*PE*	*SE*	*p*	*PE*	*SE*	*p*	*PE*	*SE*	*p*
Constant	−2.16	0.17		−2.19	0.16		−2.21	0.17		−3.21	0.39		−3.25	0.40		−3.24	0.42	
Ethnic background (Dutch = 1)	−0.24	0.18	0.20	−0.21	0.17	0.24	−0.18	0.19	0.32	0.67	0.34	0.05	0.71	0.35	0.04	0.70	0.36	0.05
Aggression toward																		
Dutch				0.35	0.05	<0.001	0.54	0.23	0.02				−0.05	0.13	0.70	−0.18	0.33	0.59
*Ethnic background (Dutch = 1)							−0.20	0.22	0.36							0.14	0.36	0.70
Turkish				0.11	0.16	0.50	0.14	0.39	0.72				0.55	0.28	0.05	0.39	0.34	0.24
*Ethnic background (Dutch = 1)							−0.05	0.50	0.93							0.27	0.57	0.63
Sex (boy = 1)	0.13	0.09	0.12	0.08	0.09	0.40	0.08	0.09	0.40	0.17	0.19	0.37	0.17	0.20	0.38	0.17	0.20	0.38
Number of Dutch classmates	Exp.			Exp.			Exp.			−0.06	0.02	0.004	−0.06	0.02	.004	−0.06	0.02	0.01
Number of Turkish classmates	−0.04	0.06	0.47	−0.03	0.05	0.60	−0.03	0.05	0.63	Exp.			Exp.			Exp.		
Variance classroom	0.12	0.06		0.11	0.05		0.11	0.05		0.36	0.17		0.35	0.17		0.35	0.17	
Log likelihood	−1573.52			−1547.40			−1546.82			−476.19			−474.34			−474.17		

The number of Dutch or Turkish classmates was used as an exposure variable to account for opportunity differences

*It indicates an interaction effect

### Aggression Toward Peers of Different Ethnic Groups

#### Rejection

Hypothesis 3 stated that aggressive adolescents would be rejected by classmates from the ethnic group to whom the victim belongs, but not by classmates from other ethnic groups in the classroom. Models 2 in Table 4 were used to test this hypothesis. Adding the variables on aggression toward Dutch and Turkish peers resulted in a small decrease of the log likelihood in Model 2 (rejection by Dutch: *Χ*^2^(2) = 5.88, *p* = 0.05; rejection by Turkish: *Χ*^2^(2) = 5.88, *p* = 0.14), indicating that the additional effects improved the model only slightly. Model 2 shows that aggression toward Dutch was related to being rejected by more Dutch classmates (*PE* = 0.35, *p* < 0.001) but not by more Turkish classmates (*PE* = −0.05, *p* = 0.70), and that aggression toward Turkish was related to being rejected by slightly more Turkish classmates (*PE* = 0.55, *p* = 0.05) but not by more Dutch classmates (*PE* = 0.11, *p* = 0.50).

Results on the interactions between aggression and adolescents’ ethnic background (Models 3 in Table 4) were used to examine hypothesis 4, stating that adolescents’ aggression toward same-ethnic peers would relate more to rejection by classmates from the ethnic group to whom the victim belongs than aggression toward cross-ethnic peers. Table 4 shows that adding the interactions did not improve the model (rejection by Dutch: *Χ*^2^(2) = 0.78, *p* = 0.68; rejection by Turkish: *Χ*^2^(2) = 0.37, *p* = 0.83). Results show that Dutch adolescents who were aggressive toward Dutch were not rejected by more Dutch classmates than Turkish adolescents who were aggressive toward Dutch (*PE* = −0.20, *p* = 0.36). Similarly, Turkish adolescents who were aggressive toward their same-ethnic Turkish peers were not found to be rejected by more same-ethnic classmates than Dutch adolescents who were aggressive toward Turkish (difference estimated = 0.27, *p* = 0.63). Thus, no support was found for hypothesis 4.

#### Friendship

Models 2 in Table 3 examined the association between aggression toward Dutch and Turkish and receiving friendship nominations from Dutch and Turkish classmates. The table shows that adding aggression to the model did not improve the model substantively (befriended by Dutch: *Χ*^*2*^(2) = 2.19, *p* = 0.33; befriended by Turkish: *Χ*^*2*^(2) = 3.90, *p* = 0.14). Results show that aggression toward Turkish peers was related to being befriended by more Dutch classmates (*PE* = 0.25, *p* = 0.02). Similarly, aggression toward Dutch peers was related to being befriended by more Turkish classmates (*PE* = 0.24, *p* = 0.001). In Models 3, interactions between aggression and adolescents’ ethnic background were added to examine whether these associations differ by adolescents’ ethnic background. Adding the interactions to the model did not improve the model (befriended by Dutch: *Χ*^*2*^(2) = 0.08, *p* = 0.96; befriended by Turkish: *Χ*^*2*^(2) = 0.89, *p* = 0.64). It was not found that the relation between aggression toward Turkish peers and being befriended by Dutch classmates differed for Dutch or Turkish adolescents. That is, Dutch adolescents who were aggressive toward their Turkish peers were not befriended by more Dutch classmates than Turkish adolescents who were aggressive toward Turkish peers (*PE* = −0.02, *p* = 0.95). For Turkish adolescents, it was also not found that they were befriended by more same-ethnic classmates when they were aggressive toward their Dutch, cross-ethnic peers (difference estimated = −0.07, *p* = 0.59). Therefore, no support was found for hypothesis 5, stating that adolescents who are aggressive toward cross-ethnic peers would be befriended by more same-ethnic classmates than cross-ethnic adolescents who are aggressive toward this cross-ethnic group.

### Additional Analyses

In Appendix 1 models of the additional analyses for friendship and rejection among Dutch-Moroccan and Dutch-Surinamese adolescents can be found. Overall, the findings on Dutch and Moroccan adolescents were in line with those for Dutch and Turkish adolescents. The results for Dutch and Surinamese adolescents were less in line with the findings for Dutch and Turkish adolescents. Whereas among Dutch and Turkish adolescents, Dutch adolescents were befriended by more Dutch classmates than Turkish adolescents, Dutch adolescents were not befriended by more Dutch classmates than Surinamese adolescents. That is, receiving friendship nominations from Dutch classmates was not related to adolescents’ ethnic background among Dutch and Surinamese adolescents. Furthermore, in contrast to hypothesis 4, it was found that Dutch adolescents who were aggressive toward Dutch peers were rejected by fewer Dutch classmates (*PE* = 0.21, *p* < 0.001) than Surinamese adolescents who were aggressive toward Dutch peers (*PE* = 0.45, *p* < 0.001). Aggression toward Dutch was thus more strongly related to rejection by Dutch classmates if the aggressor was Surinamese, i.e., cross-ethnic.

## Discussion

In the context of ethnically diverse classrooms, interethnic aggression may be perceived as behavior that reinforces ethnic boundaries. Whereas previous research suggests that adolescents' aggressive behavior in itself does not highlight ethnic boundaries, it remains unclear whether classmates’ responses to same- and cross-ethnic aggression strengthen ethnic boundaries. This article examined how adolescents’ aggression toward same- and cross-ethnic peers relates to the positive (friendship) and negative (rejection) relationship nominations they receive from same- and cross-ethnic classmates.

### The Role of Ethnicity in Friendship and Rejection

In line with previous research (Boda and Néray 2015; Leszczensky and Pink 2015) and this study’s first hypothesis, it was found that adolescents received overall more friendship nominations from same-ethnic classmates than cross-ethnic classmates. Partially in line with our second hypothesis, Dutch adolescents were only slightly more rejected by Turkish classmates than Turkish adolescents. In contrast to the hypothesis, Turkish adolescents were not found to be more rejected by Dutch classmates than Dutch classmates. These findings partially support arguments based on social identity theory, stating that in order to achieve a positive social identity, which is closely linked to individual’s group membership, individuals favor their in-group and devaluate out-groups (Tajfel 1982; Tajfel and Turner 1979). The results of this study showed that although adolescents were more likely to be befriended by same-ethnic classmates (in-group favoritism), they were generally not more likely to be rejected by cross-ethnic classmates (out-group devaluation). Based on these findings, it could be concluded that overall, ethnic boundaries are more important for adolescents’ positive peer relationships than negative peer relationships. This is in line with previous research, showing that although adolescents from different ethnic backgrounds may vary in the extent to which they have negative peer relationships, cross-ethnic negative peer relationships were not more common than same-ethnic negative peer relationships (Tolsma et al. 2013). The findings from this study also relates to the idea that same-ethnic peers are more important for adolescents’ goal pursuit than cross-ethnic peers. As youth’s positive peer relationships are more important for their goal pursuit than negative peer relationships, they will be less selective regarding negative peer relationships. Thus, youth may be less likely to direct their positive peer relationships toward cross-ethnic peers but are less likely to base their negative peer relationships on ethnic background.

### Aggression Toward Peers of Different Ethnic Groups

In line with the third hypothesis, it was found that adolescents’ aggressive behavior was related to rejection by classmates from the group that was the target of aggression. This supports the idea that individuals act as a response to their awareness of characteristics that threaten their goal pursuit by disliking these characteristics (Lindenberg 2001), such as aggression to same-ethnic peers. Further, it was found that adolescents who were aggressive toward one of the groups were not rejected by classmates from the other group. That is, aggressors of Dutch peers were not rejected by their Turkish classmates and vice versa. This is in line with the assumption that adolescents’ goal pursuit is not threatened by peers who are aggressive toward adolescents’ out-group peers (Veenstra et al. 2010).

Additionally, it was expected that adolescents’ aggression toward same-ethnic peers would more strongly relate to rejection by classmates from the ethnic group to whom the victim belongs than aggression toward cross-ethnic peers. However, in contrast to the fourth hypothesis, it was not found that the relation between adolescents’ aggression and rejection by classmates from the group that is the target of aggression was stronger if the adolescent had the same ethnic background as the victimized group. Instead, it was found that adolescents’ aggression was related to rejection by classmates from the group that was the target of aggression irrespective of adolescents’ ethnic background. Thus, adolescents’ aggression toward classmates’ same-ethnic peers was related to rejection by classmates in general, irrespective of whom the aggressor was. This suggests that aggression toward same-ethnic peers might not be perceived as betrayal by same-ethnic classmates.

Although it was found that adolescents who were aggressive toward Dutch were more befriended by Turkish classmates and vice versa, this was found irrespective of adolescents’ ethnic background. For example, both Turkish and Dutch adolescents who were aggressive toward Dutch peers were more befriended by Turkish classmates. This meant that no support was found for the fifth hypothesis that adolescents who were aggressive toward cross-ethnic peers were more befriended by same-ethnic peers. It was argued that adolescents may be rewarded by their same-ethnic classmates for being aggressive toward cross-ethnic peers because cross-ethnic aggression is a way to devaluate the out-group and consequently benefit adolescents’ positive social identity (Tajfel and Turner 1979). With the current findings, this argumentation might still hold. Irrespective of the ethnic background of the aggressor, classmates’ social identity development might benefit from aggression toward out-groups by devaluating the out-group.

Overall, these findings suggest that classmates’ positive and negative responses to adolescents were related to adolescents’ aggressive behavior based on the ethnic background of the victim, not on the ethnic background of the aggressor. Both Dutch and Turkish adolescents who were aggressive toward Dutch peers were rejected by Dutch classmates and befriended by Turkish classmates and vice versa. That is, it was not found that the ethnic composition of the aggressor-victim dyad, i.e., same- or cross-ethnic, was related to the relationship nominations adolescents receive from classmates. Instead, only the ethnic background of the victim was related to the relationship nominations adolescents receive from classmates. Ethnic boundaries in friendship and rejection were therefore reinforced by the relation between adolescents’ aggressive behavior toward same- and cross-ethnic victims and the nominations they receive from classmates but not by whether aggression in itself was cross- or same-ethnic. In the context of interventions aiming to promote integration between ethnic groups in schools, this implies that integration between ethnic groups in schools relates to aggression in general, not only to cross-ethnic aggression.

### Ethnic Group Differences

Additional analyses showed that the results among Dutch and Turkish adolescents were relatively robust. Especially among Dutch and Moroccan adolescents, the findings were largely comparable to the findings among Dutch and Turkish adolescents. For Dutch and Surinamese adolescents, however, different results were found, indicating less in- and out-group processes. These divergent results may be explained by cultural distance (Beiser et al. 2015; Lundborg 2013; Schiefer et al. 2012). In the Dutch context, Surinamese adolescents are culturally closer to Dutch adolescents than Turkish or Moroccan adolescents: they speak the same language and share similar religious beliefs. Feelings of cultural closeness are related to more positive attitudes (Berry 2003) and may therefore explain why Dutch adolescents were not more befriended by Dutch classmates than Surinamese adolescents. In contrast, Turkish and Moroccan adolescents’ culture is more distant from the Dutch culture. In drawing conclusions on the results it should be taken into account that Dutch and Turkish adolescents’ cultural backgrounds are relatively distant. For groups that are culturally less distant from each other, such as Dutch and Surinamese adolescents, in- and out-group distinctions may be less clear. Consequently, for these groups the relations between being aggressive toward and being befriended or rejected by cross-ethnic classmates may be more in line with in-group processes than out-group processes.

### Limitations and Future Directions

The present study aimed to gain insights into the role of adolescents’ ethnic background in peer friendships and rejection and how this was related to aggression toward these specific ethnic groups. The sample of this study restricted the analyses of the data to be cross-sectional because of considerable attrition between waves, especially for immigrant adolescents. Therefore, it was not possible to examine whether adolescents’ aggressive behavior affects the nominations they receive for friendship and rejection from classmates, or whether the effect goes in the opposite direction. For example, it could also be that adolescents behave aggressively toward classmates from a specific group because they were rejected by classmates from this group. Other methods, such as longitudinal social network analyses (Snijders et al. 2010), might be able to obtain a more complete picture of the relations between being aggressive toward and being befriended or rejected by same- and cross-ethnic classmates.

It was argued that aggressive behavior toward peers is a powerful means to gain status in the classroom and that, when used strategically, aggressive behavior poses a minimum risk for loss of connectedness by significant peers (Sijtsema et al. 2009; Veenstra et al. 2010). Nevertheless, the measure used to examine aggressive behavior in this study (“who is mean to you?”) captures more general forms of aggressive behavior. Being mean to someone does not only include strategic behavior to gain status, it could also include more innocent forms of aggressive behavior, such as teasing between friends. As, for example, teasing between friends might not affect the friendship nominations adolescents receive from classmates, aggressive behavior as measured in this study might have a smaller impact on friendships and rejection than pure strategic aggressive behavior. This suggests that the results might have been stronger if a more accurate measure of strategic aggressive behavior was used.

Some of the findings in this study may be explained by classroom contextual factors such as the ethnic composition and prevailing classroom norms (Veenstra et al. 2018). In classrooms with prominent ethnic boundaries, adolescents may be prone to the risk cross-ethnic aggression poses to their in-group identity. In such classrooms, classmates are likely to perceive aggression by cross-ethnic adolescents as an assault to their in-group, resulting in classmates specifically rejecting these cross-ethnic aggressors. In these classrooms, classmates may, however, not punish aggression by same-ethnic adolescents in order to avoid tensions within their ethnic group. In classrooms in which the prevailing norm is that students with different ethnic backgrounds associate with each other, however, classmates may be likely to punish adolescents’ aggression in general, not distinguishing between in-group or out-group aggression. Similarly, in classrooms with prominent ethnic boundaries, cross-ethnic aggression may be more likely to be related to being rewarded by same-ethnic classmates than in classrooms with less prominent ethnic boundaries. The effects of accounting for classroom norms on the association between aggression, friendships, and rejection is a promising avenue for future research.

As adolescents’ peer relationships change by age, the relation between adolescents’ aggressive behavior toward same- and cross-ethnic peers and the nominations they receive for friendship and rejection by classmates might change as well. For example, previous research found that boys who bullied girls were more accepted by other boys in middle childhood, but not in preadolescence (Veenstra et al. 2010). Although such changes in boy-girl interactions are more typical for adolescence than changes in same- or cross-ethnic relationships, future research may consider whether aggression toward same- and cross-ethnic peers is differently related to friendship and rejection in age groups other than studied here.

Furthermore, future research may consider the role of ethnic identity in adolescents’ response to same- and cross-ethnic aggressive behavior. Previous research has recognized the importance of ethnic identification for adolescents’ peer relationships (Knifsend et al. 2016; Syed et al. 2018), showing that stronger ethnic identities hinder opportunities for cross-ethnic positive relationships. Little is known, however, on how adolescents’ ethnic identity development influences negative peer relationships. Furthermore, in the case of Turkish adolescents in the Netherlands, immigrant adolescents’ ethnic identity is likely to be complex, relating to both their ethnic origin and their identification as Dutch. Investigating how such complex ethnic identities affect adolescents’ peer relationships and behaviors might be particularly interesting for future research.

Previous studies have argued for differentiating between various ethnic groups in studying peer relationships and processes (Bikmen 2011; Vitoroulis and Vaillancourt 2018). By investigating two specific ethnic groups and not combining multiple immigrant groups, this study aimed to take into account the possibility that processes of friendship and rejection differ between ethnic groups. Moreover, by conducting additional analyses on two other immigrant groups that differ in their cultural distance to the Dutch society, nuance was brought to the findings. The findings highlight the importance of accounting for differences between various ethnic groups in adolescents’ peer relationships. Similarly, in interpreting the results of this study it should be taken into account that this study took place in a specific context, with a long history of immigration and integration. In contexts with more recent immigration or with more prominent tensions between ethnic groups, the results may differ.

## Conclusion

As previous research found that cross-ethnic aggression was not more likely than same-ethnic aggression, aggressive behavior in itself does not seem to emphasize ethnic boundaries. It was unclear, however, whether classmates’ responses to adolescents’ same- and cross-ethnic aggression emphasize ethnic boundaries in the classroom. This study investigated how adolescents’ ethnic background influences friendships and rejection and how adolescents’ same- and cross-ethnic aggressive behavior is related to the relationship nominations they receive from classmates. Same-ethnic classmates were more likely to be friends, but adolescents were overall not more rejected by cross-ethnic than same-ethnic classmates. Moreover, adolescents’ aggression was related to rejection by classmates from the targeted group only, irrespective of adolescents’ ethnic background. Furthermore, adolescents’ aggressive behavior was related to being befriended by classmates from the group that was not the target of the aggression, irrespective of adolescents’ ethnic background. These results suggest that classmates’ positive and negative responses to adolescents are related to adolescents’ aggression based on the ethnicity of the victim, not the aggressor. This suggests that integration between ethnic groups in schools relates to aggression in general, not only to cross-ethnic aggression.

## Appendix 1

Additional analyses on Dutch and Moroccan, and Dutch and Surinamese adolescents (Table 5–8).

**Table 5:** Results of multiple multilevel Poisson and negative binomial regression models^a^ for friendship among Dutch and Moroccan adolescents (*n* = 926)

	Befriended by Dutch									Befriended by Moroccan								
	Model 1			Model 2			Model 3			Model 1			Model 2			Model 3		
Parameter	*PE*	*SE*	*p*	*PE*	*SE*	*p*	*PE*	*SE*	*p*	*PE*	*SE*	*p*	*PE*	*SE*	*p*	*PE*	*SE*	*p*
Constant	−2.40	0.20		−2.42	0.20		−2.40	0.19		−0.84	0.22		−0.85	0.21		−0.83	0.22	
Ethnic background (Dutch = 1)	0.63	0.20	0.002	0.65	0.20	0.002	0.63	0.20	0.002	−1.33	0.22	<0.001	−1.31	0.22	<0.001	−1.33	0.22	<0.001
Aggression toward																		
Dutch				−0.002	0.04	0.95	0.00	0.14	1.00				0.14	0.09	0.13	0.15	0.23	0.40
*Ethnic background (Dutch = 1)							−0.004	0.15	0.98							−0.04	0.24	0.87
Moroccan				0.17	0.11	0.14	0.02	0.51	0.97				0.02	0.14	0.91	−0.10	0.14	0.47
*Ethnic background (Dutch = 1)							0.18	0.55	0.75							0.34	0.44	0.44
Sex (boy = 1)	0.02	0.06	0.69	0.02	0.06	0.70	0.02	0.06	0.71	−0.04	0.18	0.84	−0.04	0.18	0.80	−0.04	0.17	0.81
Number of Dutch classmates	Exp.			Exp.			Exp.			0.03	0.02	0.05	0.03	0.02	0.08	0.03	0.02	0.07
Number of Moroccan classmates	0.04	0.06	0.25	0.03	0.03	0.28	0.04	0.03	0.26	Exp.			Exp.			Exp.		
Variance classroom	0.03	0.02		0.03	0.02		0.03	0.02		0.24	0.08		0.23	0.08		0.22	0.08	
Log likelihood	−1509.48			−1508.43			−1508.26			−570.67			−569.38			−569.02		

The number of Dutch or Moroccan classmates was used as an exposure variable to account for opportunity differences

^a^Being befriended by Dutch classmates was examined using multilevel Poisson regression models (test of overdispersion: *Χ*^2^ = 0.00, *p* = 0.50; test of multilevel model: *Χ*^2^ = 11.00, *p* < 0.001), being befriended by Moroccans was examined using multilevel negative binomial regression models (test of overdispersion: *Χ*^*2*^ = 3.79, *p* = 0.03; test of multilevel model: *Χ*^*2*^ = 22.90, *p* < 0.001)

*It indicates an interaction effect

**Table 6:** Results of multiple (multilevel) negative binomial regression models^a^ for rejection among Dutch and Moroccan adolescents (*n* = 926)

	Rejected by Dutch									Rejected by Moroccan								
	Model 1			Model 2			Model 3			Model 1			Model 2			Model 3		
Parameter	*PE*	*SE*	*p*	*PE*	*SE*	*p*	*PE*	*SE*	*p*	*PE*	*SE*	*p*	*PE*	*SE*	*p*	*PE*	*SE*	*p*
Constant	−2.11	0.15		−2.18	0.15		−2.21	0.17		−2.61	0.39		−2.80	0.32		−2.84	0.32	
Ethnic background (Dutch = 1)	−0.17	0.16	0.27	−0.11	0.16	0.48	−0.08	0.18	0.67	0.04	0.37	0.90	0.08	0.09	0.40	0.27	0.30	0.37
Aggression toward																		
Dutch				0.25	0.03	<0.001	0.62	0.17	<0.001				0.08	0.09	0.40	0.49	0.29	0.09
*Ethnic background (Dutch = 1)							−0.40	0.17	0.02							−0.45	0.31	0.15
Moroccan				0.22	0.18	0.22	−0.16	0.27	0.55				0.87	0.22	<0.001	0.95	0.18	<0.001
*Ethnic background (Dutch = 1)							0.53	0.34	0.12							−0.10	0.48	0.84
Sex (boy = 1)	0.06	0.09	0.47	0.03	0.09	0.72	0.04	0.09	0.68	0.28	0.19	0.046	0.37	0.19	.045	0.38	0.18	0.04
Number of Dutch classmates	Exp.			Exp.			Exp.			0.01	0.02	0.76	0.01	0.02	0.74	0.004	0.02	0.85
Number of Moroccan classmates	−0.04	0.04	0.25	−0.04	0.04	0.34	−0.03	0.04	0.45	Exp.			Exp.			Exp.		
Variance classroom	na			na			na			0.26	0.15		0.27	0.13		0.25	0.13	
Log likelihood	−1408.29			−1393.34			−1390.79			−452.07			−443.26			−442.26		

The number of Dutch or Moroccan classmates was used as an exposure variable to account for opportunity differences

^a^Being rejected by Dutch classmates was examined using single-level negative binomial regression models (test of overdispersion: *Χ*^*2*^ = 305.27, *p* < 0.001; test of multilevel model: *Χ*^*2*^ = 0.01, *p* = 0.47), being rejected by Moroccans was examined using multi-level negative binomial regression models (test of overdispersion: *Χ*^*2*^ = 2.26, *p* = 0.07; test of multilevel model: *Χ*^*2*^ = 6.99, *p* < 0.001)

*It indicates an interaction effect

**Table 7:** Results of multiple (multilevel) Poisson regression models^a^ for friendship among Dutch and Surinamese adolescents (*n* = 1219)

	Befriended by Dutch									Befriended by Surinamese								
	Model 1			Model 2			Model 3			Model 1			Model 2			Model 3		
Parameter	*PE*	*SE*	*p*	*PE*	*SE*	*p*	*PE*	*SE*	*p*	*PE*	*SE*	*p*	*PE*	*SE*	*p*	*PE*	*SE*	*p*
Constant	−1.84	0.07		−1.84	0.07		−1.84	0.07		−1.64	0.14		−1.65	0.14		−1.65	0.14	
Ethnic background (Dutch = 1)	0.002	0.07	0.98	0.003	0.07	0.97	0.01	0.08	0.87	−0.29	0.14	0.04	−0.28	0.14	0.04	−0.29	0.14	0.04
Aggression toward																		
Dutch				−0.001	0.02	0.97	−0.20	0.13	0.11				0.03	0.06	0.69	0.16	0.16	0.31
*Ethnic background (Dutch = 1)							0.20	0.13	0.11							−0.16	0.17	0.35
Surinamese				−0.12	0.07	0.08	0.32	0.34	0.34				0.19	0.14	0.18	0.13	0.38	0.73
*Ethnic background (Dutch = 1)							−0.48	0.35	0.17							0.08	0.41	0.85
Sex (boy = 1)	0.10	0.05	0.07	0.10	0.05	0.08	0.09	0.05	0.09	0.08	0.10	0.44	0.08	0.10	0.42	0.09	0.10	0.38
Number of Dutch classmates	Exp.			Exp.			Exp.			−0.02	0.01	0.09	−0.02	0.01	0.13	−0.02	0.01	0.13
Number of Surinamese classmates	−0.05	0.03	0.08	−0.05	0.03	0.10	−0.05	0.03	0.10	Exp.			Exp.			Exp.		
Variance classroom	0.01	0.01		0.01	0.01		0.01	0.01		na			na			na		
Log likelihood	−2137.03			−2136.08			−2134.09			−746.66			−745.84			−745.51		

The number of Dutch or Surinamese classmates was used as an exposure variable to account for opportunity differences

^a^Being befriended by Dutch classmates was examined using multilevel Poisson regression models (test of overdispersion: *Χ*^2^ = 0.54, *p* = 0.23; test of multilevel model: *Χ*^2^ = 4.71, *p* = 0.02), being befriended by Surinamese classmates was examined using single-level Poisson regression models (test of overdispersion: *Χ*^2^ = 0.00, *p* = 0.50; test of multilevel model: *Χ*^2^ = 0.00, *p* = 0.50)

*It indicates an interaction effect

**Table 8:** Results of multiple Poisson and negative binomial regression models^a^ for rejection among Dutch and Surinamese adolescents (*n* = 1219)

	Rejected by Dutch									Rejected by Surinamese								
	Model 1			Model 2			Model 3			Model 1			Model 2			Model 3		
Parameter	*PE*	*SE*	*p*	*PE*	*SE*	*p*	*PE*	*SE*	*p*	*PE*	*SE*	*p*	*PE*	*SE*	*p*	*PE*	*SE*	*p*
Constant	−2.49	0.14		−2.51	0.13		−2.54	0.14		−2.53	0.26		−2.59	0.27		−2.55	0.26	
Ethnic background (Dutch = 1)	0.24	0.14	0.09	0.25	0.14	0.07	0.27	0.15	0.06	0.34	0.26	0.19	0.34	0.26	0.20	0.29	0.25	0.26
Aggression toward																		
Dutch				0.23	0.04	<0.001	0.45	0.11	<0.001				0.05	0.07	0.45	−0.36	0.48	0.46
*Ethnic background (Dutch = 1)							−0.24	0.12	0.04							0.42	0.49	0.39
Surinamese				0.19	0.15	0.21	−0.06	0.42	0.89				0.63	0.12	<0.001	−0.37	0.79	0.64
*Ethnic background (Dutch = 1)							0.27	0.45	0.54							1.04	0.79	0.19
Sex (boy = 1)	−0.07	0.08	0.40	−0.10	0.09	0.26	−0.09	0.09	0.30	−0.01	0.14	0.95	0.03	0.14	0.85	0.03	0.14	0.84
Number of Dutch classmates	Exp.			Exp.			Exp.			−0.01	0.01	0.69	−0.001	0.02	0.96	−0.002	0.02	0.88
Number of Surinamese classmates	0.02	0.04	0.60	0.01	0.04	0.75	0.01	0.04	0.77	Exp.			Exp.			Exp.		
Variance classroom	na			na			na			na			na			na		
Log likelihood	−1889.76			−1876.39			−1875.73			−600.32			−590.63			−588.85		

The number of Dutch or Surinamese classmates was used as an exposure variable to account for opportunity differences

^a^Being rejected by Dutch classmates was examined using single-level negative binomial regression models (test of overdispersion: *Χ*^2^ = 704.50, *p* < 0.001; test of multilevel model: *Χ*^2^ = 0.78, *p* = 0.19), being rejected by Surinamese classmates was examined using single-level Poisson regression models (test of overdispersion: *Χ*^*2*^ = 0.20, *p* = 0.33; test of multilevel model: *Χ*^*2*^ = 0.45, *p* = 0.25)

*It indicates an interaction effect

## References

[CR1] Beiser M, Puente-Duran S, Hou F (2015). Cultural distance and emotional problems among immigrant and refugee youth in Canada: Findings from the New Canadian Child and Youth Study (NCCYS). International Journal of Intercultural Relations.

[CR2] Berry JW (2003). A psychology of immigration. Journal of Social Issues.

[CR3] Bikmen N (2011). Asymmetrical effects of contact between minority groups: Asian and Black students in a small college. Cultural Diversity and Ethnic Minority Psychology.

[CR4] Boda Z, Néray B (2015). Inter-ethnic friendship and negative ties in secondary school. Social Networks.

[CR5] Cameron AC, Trivedi PK (2009). Microeconometrics using Stata.

[CR6] Cameron AC, Trivedi PK (2013). Regression analysis of count data (2nd edn).

[CR7] Dollmann, J., Jacob, K., & Kalter, F. (2014). *Examining the diversity of youth in Europe. A classification of generations and ethnic origins using CILS4EU data*. www.mzes.uni-mannheim.de

[CR8] Ganzeboom HBG, De Graaf PM, Treiman DJ, De Leeuw J (1992). A standard international socio-economic index of occupational status. Social Science Research.

[CR9] Hamm JV (2000). Do birds of a feather flock together? The variable bases for African American, Asian American, and European American adolescents’ selection of similar friends. Developmental Psychology.

[CR11] Ibarra H (1992). Homophily and differential returns: sex differences in network structure and access in an advertising firm. Administrative Science Quarterly.

[CR12] Kalter F, Heath AF, Hewstone M, Jonsson JO, Kalmijn M, Kogan I, Van Tubergen F (2016). Children of immigrants longitudinal survey in Four European countries (CILS4EU) – Reduced version. (ZA5656 Data file Version 1.2.0).

[CR13] Knifsend CA, Bell AN, Juvonen J (2016). Identification with multiple groups in multiethnic middle schools: What predicts social ingroup overlap?. Journal of Youth and Adolescence.

[CR14] Leszczensky L, Pink S (2015). Ethnic segregation of friendship networks in school: Testing a rational-choice argument of differences in ethnic homophily between classroom- and grade-level networks. Social Networks.

[CR15] Lindenberg S, Turner JH (2001). Social rationality versus rational egoism. Handbook of sociological theory.

[CR16] Long JS, Freese J (2006). Regression models for categorical variables using stata.

[CR17] Lundborg P (2013). Refugees’ employment integration in Sweden: cultural distance and labor market performance. Review of International Economics.

[CR18] Muthen BO, Satorra A (1995). Complex sample data in structural equation modeling. Sociological Methodology.

[CR20] Rivera MT, Soderstrom SB, Uzzi B (2010). Dynamics of dyads in social networks: Assortative, relational, and proximity mechanisms. Annual Review of Sociology.

[CR21] Schiefer D, Möllering A, Daniel E (2012). Cultural value fit of immigrant and minority adolescents: the role of acculturation orientations. International Journal of Intercultural Relations.

[CR22] Sentse M, Dijkstra JK, Salmivalli C, Cillessen AHN (2013). The dynamics of friendships and victimization in adolescence: a longitudinal social network perspective. Aggressive Behavior.

[CR23] Sijtsema JJ, Veenstra R, Lindenberg S, Salmivalli C (2009). Empirical test of bullies’ status goals: assessing direct goals, aggression, and prestige. Aggressive Behavior.

[CR24] Snijders TAB, Van de Bunt GG, Steglich CEG (2010). Introduction to stochastic actor-based models for network dynamics. Social Networks.

[CR25] Stark TH, Flache A (2012). The double edge of common interest: Ethnic segregation as an unintended byproduct of opinion homophily. Sociology of Education.

[CR26] StataCorp. (2017). Stata statistical software: release 15.

[CR27] Statistics Netherlands (2018). StatLine - Bevolking; kerncijfers. Retrieved August 9, 2019, from https://opendata.cbs.nl/statline/#/CBS/nl/dataset/37296ned/table?dl=1989F

[CR28] Strohmeier D, Spiel C, Gradinger P (2008). Social relationships in multicultural schools: Bullying and victimization. European Journal of Developmental Psychology.

[CR29] Syed M, Juang LP, Svensson Y (2018). Toward a new understanding of ethnic-racial settings for ethnic-racial identity development. Journal of Research on Adolescence.

[CR30] Tajfel H (1982). Social psychology of intergroup relations. Annual Review of Psychology.

[CR31] Tajfel H, Turner J, Austin WG, Worchel S (1979). An integrative theory of intergroup conflict. The Social Psychology of Intergroup Relations.

[CR32] Tolsma J, Van Deurzen I, Stark TH, Veenstra R (2013). Who is bullying whom in ethnically diverse primary schools? Exploring links between bullying, ethnicity, and ethnic diversity in Dutch primary schools. Social Networks.

[CR33] Travaglino GA, Abrams D, Randsley de Moura G, Marques JM, Pinto IR (2014). How groups react to disloyalty in the context of intergroup competition: Evaluations of group deserters and defectors. Journal of Experimental Social Psychology.

[CR34] Van der Ploeg R, Steglich C, Salmivalli C, Veenstra R (2015). The intensity of victimization: associations with children’s psychosocial well-being and social standing in the classroom. PLoS ONE.

[CR35] Veenstra R, Dijkstra JK, Kreager DA, Bukowski WM, Laursen B, Rubin KH (2018). Pathways, networks, and norms: A sociological perspective on peer research. Handbook of peer interactions, relationships, and groups.

[CR36] Veenstra R, Lindenberg S, Munniksma A, Dijkstra JK (2010). The complex relation between bullying, victimization, acceptance, and rejection: Giving special attention to status, affection, and sex differences. Child Development.

[CR37] Vitoroulis I, Vaillancourt T (2018). Ethnic group differences in bullying perpetration: a meta-analysis. Journal of Research on Adolescence.

